# An Assessment on Ethanol-Blended Gasoline/Diesel Fuels on Cancer Risk and Mortality

**DOI:** 10.3390/ijerph18136930

**Published:** 2021-06-28

**Authors:** Steffen Mueller, Gail Dennison, Shujun Liu

**Affiliations:** 1 Energy Resources Center, The University of Illinois at Chicago, Chicago, IL 60607, USA; muellers@uic.edu; 2 The Hormel Institute, University of Minnesota, Austin, MN 55912, USA; gaildenn@umn.edu

**Keywords:** biofuels, benzene, BTEX, BTX, COVID-19, carcinogens, DNA methyltransferases, DNMT, DNA methylation, ethanol, epigenotoxicity, gasoline combustion, genotoxicity, histone modification, HAT, histone acetyltransferases, histone deacetylases, HDAC, microRNAs, PAHs, PM emission, ten–eleven translocation methylcytosine dioxygenases, TET

## Abstract

Although cancer is traditionally considered a genetic disease, the epigenetic abnormalities, including DNA hypermethylation, histone deacetylation, and/or microRNA dysregulation, have been demonstrated as a hallmark of cancer. Compared with gene mutations, aberrant epigenetic changes occur more frequently, and cellular epigenome is more susceptible to change by environmental factors. Excess cancer risks are positively associated with exposure to occupational and environmental chemical carcinogens, including those from gasoline combustion exhausted in vehicles. Of note, previous studies proposed particulate matter index (PMI) as a measure for gasoline sooting tendency, and showed that, compared with the other molecules in gasoline, 1,2,4–Trimethylbenzene, 2–methylnaphthalene and toluene significantly contribute to PMI of the gasoline blends. Mechanistically, both epigenome and genome are important in carcinogenicity, and the genotoxicity of chemical agents has been thoroughly studied. However, less effort has been put into studying the epigenotoxicity. Moreover, as the blending of ethanol into gasoline substitutes for carcinogens, like benzene, toluene, xylene, butadiene, and polycyclic aromatic hydrocarbons, etc., a reduction of secondary aromatics has been achieved in the atmosphere. This may lead to diminished cancer initiation and progression through altered cellular epigenetic landscape. The present review summarizes the most important findings in the literature on the association between exposures to carcinogens from gasoline combustion, cancer epigenetics and the potential epigenetic impacts of biofuels.

## 1. Introduction

Air pollution, containing harmful or poisonous substances, is a worldwide threat to human health, even at low doses. A major source of air pollution in urban areas is the combustion of diesel and gasoline fuels emitting >75% of atmospheric pollutants [1,2,3]. A considerable and growing literature demonstrates that human exposure to transportation-related pollutants causes many cancerous and noncancerous diseases, such as cardiopulmonary aberrations, reproductive dysfunction, neurodegenerative disorders, leukemia and lung cancer [2], thus increasing mortality and morbidity rates. Pollutants from transportation (Table 1) are a complex mixture of gaseous and solid components, including carbon monoxide, carbon dioxide, nitrogen oxides, volatile organic compounds (VOCs), polycyclic aromatic hydrocarbons (PAHs), secondary reaction products, and particulate matter (PM), and others [4]. Emissions from gasoline vehicles were selected for the major topic in this review. Gasoline constitutes the largest share of transportation fuels. It contains over 250 different hydrocarbons, and the defined adjustments to the hydrocarbon mix of the fuel will change the emissions profile and thereby the health impact of the fuel, which we consider in this paper.

Many hydrocarbons in gasoline fuel are volatile organic compounds that are added to give gasoline well-defined combustion properties in engines. A subgroup of those volatile organic compounds, the aromatic hydrocarbons (e.g., benzene, toluene, xylene, ethylbenzene), are added to gasoline fuel due to their high octane rating, which prevents engines from premature combustion (knocking) and damage. Aromatics, however, are a large source of PM emissions via two principal pathways: a) incomplete combustion leads to soot emissions from the tailpipe, and b) secondary organic aerosol formation, which in turn contributes to ultrafine particle formation in the PM_2.5_ (less than 2.5 micrometers in size) category. Aromatic compounds of low molecular weight like benzene, toluene, and xylene contribute to indirect PM emissions. A subgroup of aromatics, the polycyclic aromatic hydrocarbons (PAHs) contribute to direct PM formation if they are of low molecular weight, while their high molecular weight components form indirect PM.

PM emission assessments from gasoline have recently received greater attention, since modern gasoline direct-injection engines (GDI) show an increase in that emissions group. In many countries including the U.S., ethanol produced from corn, sugarcane, or cellulosic materials is being increasingly added to gasoline. Ethanol has a very high-octane rating and it therefore substitutes and dilutes aromatics in gasoline. Thus, replacing aromatics with ethanol is generally shown to reduce PM emissions [1]. Conversely, some studies have shown that ethanol can lead to an increase in acetaldehyde emissions. The cancer risks due to the reduced PM and PAH emissions from aromatics substitution with ethanol while considering a potential increase in acetaldehyde are a focus of this review.

PM has been documented as a human carcinogen (group I, IARC, 2013) [5]. Transportation-derived PM increases the incidence of human diseases [6,7], and cohort studies in the U.S. and Europe have found an association of exposure to transportation-derived PM with cardiopulmonary-related diseases and cancers [8,9,10,11,12]. PAHs display toxicity and mutagenicity [13,14]. Gasoline and diesel emissions are different in their carcinogenic PAHs that are widespread environmental contaminants from incomplete combustion of organic materials. The International Agency for Research on Cancer (IARC) has classified diesel engine emissions as carcinogenic to humans (Group 1), and gasoline engine emissions as a possible carcinogen to humans (Group 2B) [1]. As many health outcomes have not been examined, there is clearly a need for more thorough evaluation of the impacts of gasoline exhaust on transportation-related health effects.

Epidemiological and experimental studies suggested that exposure to chemicals from gasoline exhaust increases the incidence of multiple cancers [15,16,17,18,19,20,21,22], like hematologic malignancies, lung cancer, or prostate cancer. Mechanistically, the epigenome and genome may be equally important in carcinogenicity, but the genotoxicity of chemical agents and exposure-related transcriptomic responses have been more comprehensively investigated. Compared with genetic changes, epigenetic modifications are more susceptible to change by environmental stimuli (e.g., air pollutants) and arise rapidly. Further, epigenetic alterations are early indicators of genotoxic and non-genotoxic carcinogen exposure. Thus, epigenetic mechanisms may be more reasonable to explain how environmental chemicals induce cancer and other diseases. To date, DNA methylation aberrations are the most commonly studied, followed by abnormal changes of non-coding RNAs and histone modifications [23]. However, there exists a debated literature regarding the carcinogenic potential of air pollutants derived from gasoline combustion, and the epigenetic alterations of air pollutants-associated human malignancies. Importantly, research has shown that blending ethanol into gasoline and its indirect substitution effect on harmful carcinogens benefits human beings, including a likely decrease of cancer risk and occurrence. However, almost all studies regarding ethanol’s direct health effects and the underlying molecular mechanisms focus on ethanol/alcohol drinking/consumption. Therefore, much of the existing literature on ethanol’s impacts on toxicity and epigenetics is not relevant or appropriate for comparisons with the effects of exposure to gasoline combustion emissions. Because the dosages and concentration of ethanol emissions (from gasoline) inhalation are really low, the underlying epigenetic base could be totally different or even opposite when compared to those underlying ethanol drinking/consumptions. The purpose of this review is to describe the crucial aspects of epigenetic aberrations, and to outline the ways in which environmental chemicals can affect this cancer hallmark. The overall aim was to make scientists aware of (1) the increasing need to delineate the underlying mechanisms via which chemicals at low doses can induce epigenetic changes, thus promoting carcinogenesis; (2) the potential benefits and the underlying molecular mechanisms of blending ethanol into gasoline; and (3) the different or even opposite outcomes obtained from chronic/heavy alcohol drinking studies when compared with ethanol inhalation.

## 2. Carcinogenic Potential of Chemicals Associated with Air Pollution from Gasoline

The chemical mixture emitting from gasoline combustion that constitutes the main carcinogenic concerns consists of benzene, toluene, xylene, butadiene, 1,2,4–Trimethylbenzene, 2–methylnaphthalene, acetaldehyde, and many PAHs (Table 1). In general, epidemiological and experimental evidence supports the carcinogenic potential of these chemicals, even exposure to low doses [15,16,17,18,19,20,21,22]. Among them, benzene, presented in gasoline (1% by volume), is widely known as one of the predominant air pollutants in the environment, particularly in proximity to gas stations and in areas of high vehicular traffic [24], and has attracted the most attention. Benzene has been classified as a group 1A carcinogen [25]. Findings from many studies support that workers exposed to benzene have higher incidence of hematological malignancies, primarily acute myeloid leukemia (AML), chronic lymphocytic leukemia (CLL), and myelodysplastic syndrome (MDS) [26,27,28,29,30,31,32,33,34,35,36,37,38,39,40,41,42,43]. There is some limited evidence showing that benzene exposure has been causatively linked with increased risk of lung cancer [44,45,46], breast cancer (in animal model) [47,48,49], prostate cancer [50], kidney cancer [51], or bladder cancer [52]. Some studies on humans have shown that benzene is one of the risk factors for the development of breast cancer [24]. A case-control study by Petralia et al. [53] indicated an association between breast cancer risk and occupational exposure to benzene in women. In addition, Costantini et al. [54] conducted an epidemiological cohort study of female workers using benzene-based glues in a shoe factory in Italy. Their findings suggested that chronic exposure to benzene can be one of the risk factors for breast cancer. Thus, there is adequate evidence supporting that benzene is carcinogenic to human.

Further studies also examined the role of butadiene, toluene, xylene, 1,2,4–trimethylbenzene, acetaldehyde, 2–methylnaphthalene and many PAHs emitting from gasoline exhaustion in cancer incidence and mortality. Limited studies in a population-based investigation showed that occupational exposure to one or more of these agents (butadiene, toluene, xylene) may be associated with lung cancer, higher risks of overall prostate cancer and an increased risk of hematological malignancies [16,18,19]. As a single agent, studies in workers and animals exposed to toluene generally suggest that toluene and xylene may not be carcinogenic [55]. The International Agency for Research on Cancer (IARC) determined that toluene is not classifiable as to its carcinogenicity in humans. While no direct human evidence is available, there is recent evidence of carcinogenicity of toluene and xylene at high concentrations in experimental animals. Limited studies provide evidence of an association of occupational exposure to toluene and the risk of bladder cancer [52]. However, several studies did suggest that exposure to 1,3–butadiene (emissions) is associated with excess cancer risks, including hematological malignancies, in human and mouse models [56,57,58,59,60]. Regarding the PAHs that are currently classified as human carcinogens, excessive exposure to PAHs often results in elevated incidence of cancers [61], such as lung cancer [21,62], a disease with the highest cancer mortality, and bladder cancer [62], even functions as a key cofactor in HPV-mediated carcinogenesis [63]. Finally, a couple of studies revealed that 1,2,4–trimethylbenzene, acetaldehyde and 2–methylnaphthalene are carcinogenesis in squamous epithelium [22,64,65,66]. Under the cancer guidelines (2005) of the U.S. Environmental Protection Agency (EPA), the human and animal data are insufficient to determine the carcinogenic potential of 1,2,4–trimethylbenzene in humans. Further, based on the Agency for Toxic Substances and Disease Registry (ATSDR), there is no direct evidence in humans that naphthalene and 2–methylnaphthalene can induce cancerous transformation, although some studies showed that exposure to 2–methylnaphthalene leads to pulmonary alveolar proteinosis but does not possess unequivocal carcinogenic potential in B6C3Fj mice [67]. Notably, any future epidemiological observations of cancer risks that are associated with toluene or xylene exposure should take into consideration the suspected effects of benzene impurities. Finally, findings from animal studies should be interpreted cautiously. We should be aware that differences may exist among animal species, and between animals and humans, in the metabolism of, and sensitivity to, xylene. Conditions of exposure to xylene in animal and human studies, both occupational and experimental, are usually different.

## 3. The Positive Effects on Human Health of Blending Ethanol into Gasoline

Gasoline contains a large amount of added aromatic hydrocarbons, because these chemicals have relatively high-octane values, thereby serving as anti-knock agents in vehicle engines. Certainly, some aromatics are toxic compounds. Further, combustion emissions account for more than 50% of fine particle PM_2.5_ air pollution and most of the primary particulate organic matter [2]. Gasoline combustion emissions are a ubiquitous source of exposure to complex mixtures of PM and non-PM pollutants. Human exposure to combustion emissions has been studied in populations in developed countries, like Europe, Japan, and the United States, and increasingly in developing countries, like China, Brazil, and Argentina. The findings have identified many mutagenic and carcinogenic chemicals [2]. Due to the severe health impacts of air pollutants from transportation emissions, developing and finding alternative fuel sources to reduce the vehicular emissions have been hot topics. Robust evidence indicates that blending ethanol into gasoline is beneficial, because: (1) ethanol does not have aromatic compounds. It therefore substitutes and dilutes aromatics in gasoline. The U.S. EPA has shown that ethanol use (biofuels) substitutes for a host of toxic aromatics in gasoline; (2) ethanol has a higher octane number than gasoline, which can improve the energy efficiency; (3) ethanol alters the distillation curve leading to an adjustment of the distillation properties of the fuel. This effect further reduces the formation of toxic emissions in a vehicle. Ethanol volumetrically dilutes multiple harmful gasoline compounds (e.g., benzene, toluene, xylene, butadiene, and polycyclic aromatic hydrocarbons, etc.) [68]; (4) Previous studies [69] revealed that an increase in the ethanol content in the fuel blends reduces the emissions of some regulated gases, carbon monoxide (CO) and total hydrocarbons (THC); and (5) emerging evidence suggests that by blending ethanol into the gasoline, all PHAs are decreased with more reductions when ethanol blending is higher [70]. Such positive impacts are further supported by the findings of Munoz et al. [70], showing that ethanol blending reduces genotoxic emissions. For example, compared with that of E0, particle number emissions with E10 and E85 are lowered by 97 and 96%; CO emission is decreased by 81 and 87%; emission of selected PAHs is lowered by 67–96% with E10 and by 82–96% with E85, and the genotoxic potentials drop by 72 and 83%, respectively. Given that air pollutants emitted from gasoline combustion have carcinogenic potential, remarkable reduction of air pollutants by biofuels could decrease the risk and occurrence of human cancers. Another benefit of biofuels is the reduction of COVID-19 infection, because recent studies and analysis [71,72,73] showed that exposure to PM could increase the susceptibility and severity of COVID-19 patient symptoms, for example, an increase of only 1 μg/m^3^ in PM_2.5_ is associated with an 8% increase in the COVID-19 death rate, and there is a positive correlation between exposure to PM and COVID-19 virus spread [74,75]. However, the molecular mechanisms to explain such positive association of PM with COVID-19 are lacking. Finally, although biofuels have ethanol vapor in the air (usually very low ethanol concentrations), this may not have negative effects on human health, because a clinical trial (NCT04554433) suggests that breathing ethanol could be beneficial for patients with COVID-19, as it reduces surface tension on the alveoli and markedly decreases sputum formation. The inflammatory and dangerous effects on patients can be controlled by the actual used concentrations.

## 4. Overview of Epigenetic Mechanisms in Cancer Development and Progression

Typical epigenetic mechanisms include DNA methylation, histone modifications (acetylation, methylation, phosphorylation, etc.) and microRNA expression, which critically regulate expression of oncogenes and tumor suppressor genes (TSGs) [76,77,78,79,80]. The concept that aberrant epigenetics is a key regulator for cancer initiation, development, maintenance and progression has been undoubtedly established, which has also been thoroughly and systematically reviewed [76,77,81,82,83,84,85,86,87,88,89,90,91]. As such, this review mainly focuses on the findings from our groups and the molecular mechanisms by which the abundance of epigenetic modulators are abnormally regulated in cancers. Further, benzene is the most broadly studied chemical in air pollutants, and it is clearly considered a carcinogen, inducing leukemia, breast cancer and lung cancer. Thus, we will mainly discuss the contribution of aberrant epigenetics to the pathogenesis of leukemia, lung cancer and breast cancer.

### 4.1. DNA Methylation and Cancer

DNA methylation involves a covalent chemical modification of DNA, which is installed mainly by DNA methyltransferase (DNMT) 1, 3a and 3b. In the presence of S–adenosyl–methionine (SAM) that serves as a methyl donor, a methyl group is added by DNMTs to the C–5 position of cytosine residues, yielding 5–methylcytosine (5mC). In general, DNMT 3a and 3b are mainly de novo enzymes, while DNMT 1 acts as both maintenance DNMT, which propagates the methylation patterns to the daughter cells (through cell division), and de novo DNMT (non-cell cycle activity) that initiates DNA methylation in cancer cells [92,93,94]. DNMTs are overexpressed [95,96,97,98], and TSGs are frequently silenced via promoter DNA hypermethylation in cancers [99,100]. In addition, DNA methylation binding proteins critically regulate DNA methylation dynamics in controlling target expression whereby cancer pathogenesis [101,102,103]. Because TSGs are master regulators of cell proliferation and survival, silencing of TSG may confer a significant advantage to cancer growth [95,96,104,105,106], as supported by the fact that TSG silencing predicts poor prognosis in cancers [95,96,97,100,104,106,107,108,109,110,111]. Importantly, DNA methylation, particularly CpG hypermethylation at TSG promoters, arises at the early stage of cancer or at the “pre-tumorigenic” phase, and the DNA methylation levels are increased in accordance with cancer development and progression. Concomitantly, aberrant DNA methylation serves as a key hallmark of cancers, including leukemia [98,100,107,112,113,114], breast cancer (luminal types, HER2 overexpressing, basal-like, etc.) [112,113,114,115,116], and lung cancer [117,118,119,120].

Due to gene overexpression or mutations in cancers [105,112,113,121,122], DNA methyltransferases (DNMTs) become hyperactive, leading to DNA hypermethylation in the promoters of TSGs. For example, DNMT 3a mutations are prevalent in cancers and such mutations decrease DNA methylation levels, which is linked with higher relapse rates and an inferior overall survival, and promotes transformation of hematopoietic cells [123,124,125,126,127]. Further, changes in DNMT gene levels are in parallel with DNA methylation amount [96,97,98,106,111]. On the other hand, once methylated, the modified cytosine (5mC) can go through a stepwise methylcytosine dioxygenase-mediated oxidation process to form 5–hydroxymethylcytosine (5hmC), 5–formylcytosine (5fC) and 5–carboxylcytosine (5caC) [128,129,130,131], promoting locus-specific reversal of DNA methylation. The ten–eleven translocation (TET) methylcytosine dioxygenases (TET1/2/3) catalyze the conversion of 5mC to 5hmC, resulting in active and passive DNA demethylation [132,133,134,135,136]. Analogous to DNA methyltransferases (DNMTs), the enzymatic activities of TETs are subjected to be regulated by gene mutations (i.e., TET 2) [137,138,139,140] and abnormal gene expression [141,142]. Such dysregulated TET activities are strongly associated with cancer pathogenesis [137,141,143]. In general, TETs serve as TSGs, as supported by showing that levels of TET expression and 5hmC are decreased in a wide range of cancers [140,142,144,145]. However, emerging evidence also supports an oncogenic role of TETs [140], as supported by the fact that TET1 is upregulated in adenocarcinoma and squamous cell carcinomas [146], and reduction of 5hmC content is associated with decreased survival rate [147]. While TET (i.e., TET2) mutations frequently occur in hematological cancers [128,148,149], missense and truncating mutations in the TET genes are also observed in solid tumors. However, the mutation rate is relatively low (0.1–10%) [128], suggesting that changes in TET gene expression are more essential in determining TET functions in solid cancers.

Although it is well appreciated that aberrant expression of DNA methyltransferases (DNMTs) and ten–eleven translocation methylcytosine dioxygenases (TETs) induces aggressive breast cancer [112,113,114,115,116], lung cancer [96,106] and leukemia, etc. [97,98,109,110,111,141], how they are dysregulated in cancers [140,141,150] is largely unclear. Thus, investigations of the molecular mechanisms involved in gene dysregulation have been always active. Our studies revealed that abnormal DNMT expression may be attributed to cell-autonomous signaling, including microRNAs [98,100], nucleolin [98], Sp1/NFkB (nuclear factor kappa B) [107], AML1/ETO [114], cytokines [104], and/or protein kinases, etc. [96,110]. Importantly, we demonstrated that upregulation of fatty acid-binding protein 4 (FABP4) by environmental stimuli (i.e., high-fat diet, obesity) upregulates DNMT1, but not DNMT3a and DNMT3b, partially through activation of the IL–6/STAT3 signaling in cell non-autonomous manner in leukemia cells [112]. Further, environmental stress mediated by chemotherapy and molecular-targeted therapy also changes DNMT gene expression [99,106,108,110]. In agreement with the outcomes from cancer therapies, a few studies showed that downregulation of TET1 expression or upregulation of DNMT1 by air pollutants is observed, resulting in DNA hypermethylation and TSG silencing [151,152,153]. However, thorough investigations are necessary to address whether and how environmental chemicals emitting from gasoline, particularly biofuels, contribute to abnormal expression of DNMTs and TETs in cancers.

### 4.2. Histone Modifications and Cancers

Histone posttranslational modifications provide a fundamental way to regulate chromatin structure, thus affecting gene transcription, DNA damage repair, DNA replication, and other cellular processes. These many modifications include phosphorylation [154,155,156], ubiquitination [157,158], methylation [159,160,161,162,163], acetylation [164,165,166], and so on. The essential roles of these modifications in tumorigenesis and cancer metastasis have been well documented and widely reviewed [167,168,169,170]. Among them, the deacetylation and acetylation of histones have attracted the most attention in understanding the causes of cancers, identifying diagnostic, prognostic, and therapeutic biomarkers, as well as developing reagents for cancer therapies.

Histone acetylation is determined by a balanced activity of histone acetyltransferases (HATs) and histone deacetylases (HDACs). HATs are enzymes that acetylate conserved lysine amino acids by transferring an acetyl group onto lysine residues of histone protein, forming ε–N–acetyl lysine. However, histone deacetylation is achieved by HDACs, including HDAC 1–11 and SIRT1–7. Altered expression and mutations of HDACs or HATs change their catalytic activities [171,172,173,174], resulting in an imbalance of histone acetylation and deacetylation, whereby aberrant expression of oncogenes or TSGs leading to cancer initiation, development, and progression. Because dysfunction of HDACs and HATs by aberrant gene expression serves as diagnostic and prognostic biomarkers [175], multiple HDAC inhibitors have entered into clinic, favorable outcomes have been achieved [176,177,178]. However, as a single agent, HDAC inhibitors have shown limited therapeutic efficacy, supporting a combination therapy with other reagents, like DNA methylation inhibitors, for a better management of cancers [179,180]. Further, the catalytic activities of HATs have been reported to be hyperactive in many human diseases ranging from cancer and inflammatory diseases to neurological disorders, through enhanced acetylation of histone or non-histone proteins [181,182]. Thus, HAT inhibitors [183,184,185], like bisubstrate inhibitors, natural product derivatives and small molecules, have been developed [186]. Despite their therapeutic potential, gaps remain between the biological outcomes of inhibitors from in vitro, even animal, studies and their potential use as therapeutic reagents in human patients. As the altered activities of HDACs and HATs from abnormal gene expression are more essential in determining cancer cell fate, further elucidating the molecular mechanisms by which these enzymes are dysregulated in cancer cells may advance our understanding of cancer and developing new inhibitors.

### 4.3. MicroRNAs and Cancers

MicroRNAs, initially discovered in 1993 [187], are small RNAs (containing about 22 nucleotides) without coding regions, and in general, function as negative gene regulators [188]. However, microRNAs are indeed similar to coding genes, which are transcribed by RNA polymerase II as long primary transcripts characterized by hairpin structures (pri–microRNAs), and processed into the nucleus by RNAse III Drosha into 70–100 nts-long pre–miRs [189]. As microRNA regulatory mechanisms are frequently altered in human cancers, abnormal expression of microRNAs is prevalent in all cancers [190]. First, analogous to protein-coding genes, ours and other studies demonstrate that microRNAs are transcriptionally regulated by genetic and/or epigenetic mechanisms, including chromosomal abnormalities (AML1–ETO) [191,192,193], SP1/NFkB [194,195], histone deacetylases (HDACs) [194,196,197], DNA methyltransferases (DNMTs) [198,199], and/or environmental factors (diet, lifestyle, fine particulate air pollution) [200,201,202,203] and many others. For example, the microRNA–34 family was downregulated by a mechanism that involves promoter DNA methylation [204,205,206]. Although not as frequently as dysregulation, microRNAs are also subjected to the regulation of mutations or deletion [207,208]. Second, miR dysfunction crucially regulates tumor growth and cancer metastasis [100,193,207,208]. To date, the role of microRNAs in cancer pathogenesis and drug resistance has been well documented and broadly reviewed [82,84,188,194]. Many microRNAs (i.e., microRNA–21, microRNA–155, microRNA–19a) function like oncogenes (oncomiRs) [188,209,210], enhancing tumorigenesis and cancer metastasis when overexpressed [210]; other microRNAs (i.e., microRNA–15, microRNA–16, microRNA–29b) [211] are tumor suppressors losing expression or functions in cancer cells [100,194,208,212]. Further, certain microRNAs (microRNA–22) have dual activities promoting or inhibiting tumor growth [213]. The critical contribution of microRNA deregulation to cancers is further strengthened by the facts that aberrant expression of microRNAs serves as potential biomarkers for cancer diagnosis, prognosis and therapeutic targets [100,194], and microRNAs themselves or their anti-nucleotides have been used to develop cancer therapeutics [83,214]. Mechanistically, microRNAs regulate cancer cell survival and proliferation by posttranscriptionally and negatively controlling their target gene expression. These cellular processes occur through binding of microRNAs to the 3′–untranslated regions (3’–UTR) of their targets (oncogenes, TSGs) [100,188], resulting in translational inhibition or degradation of target mRNAs, thereby suppressing gene expression. Notably, one microRNA can bind to more than one species of mRNA target, or multiple species of microRNAs can bind to the same mRNA targets [215], supporting the complexity of microRNAs-initiated regulation of genes whereby cancerous diseases.

**Figure 1:**
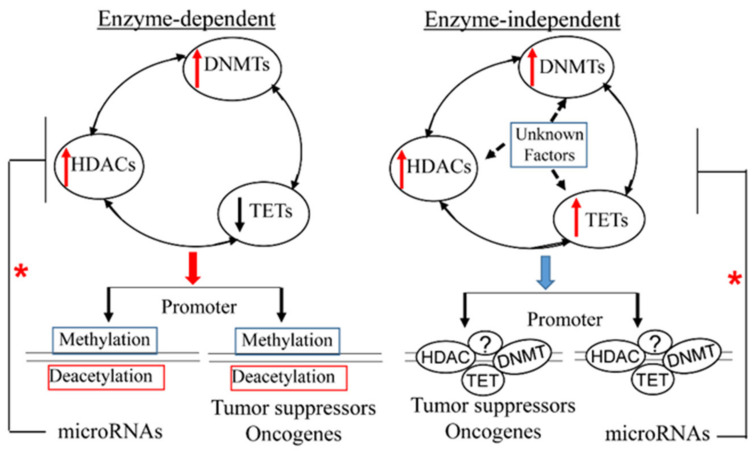
Crosstalk among epigenetic regulators determines cancer cell fate. Left: HDACs, DNMTs and TETs cooperatively regulate DNA methylation and his-tone modification change target expression; Right: HDACs, DNMTs, TETs and unknown factors form complex binding target promoters controlling their levels; * microRNA deregulation feedback to inhibit epigenetic regulators.?, unknown factor; ↑ upregulation; ↓ downregulation; ┤ Inhibition.

It is worth noting that aberrant DNA methylation, abnormal histone modifications and microRNA dysregulation do not function individually, but cooperatively contribute to the development, maintenance and progression of cancers [216,217,218]. As shown in Figure 1, this functional cooperation may result from the reciprocal regulation of their expression levels [218], for example, microRNA–DNA methylation loop [219,220], and interplay between microRNAs and histone deacetylases (HDACs) [221]. Further, in addition to having enzymatic activities, all DNA methyltransferases (DNMTs), histone deacetylases (HDACs) and ten–eleven translocation methylcytosine dioxygenases (TETs) have enzyme-independent functions. For instance, TET and DNMT or HDAC form a complex that binds target promoters up- or down-regulating target gene expression [222,223]. HDAC and DNMT form a complex through protein physical interaction to induce chromatin remodeling, thus altering gene expression [224]. Moreover, DNMT and HDAC together are also recruited by other transcriptional factors, such as AML1–ETO, to repress target gene expression [111,225,226]. Given that cancers are systematic diseases, and as many genes/pathways are involved in the initiation and progression of cancers simultaneously, future studies may need to focus more on the interplay/cooperation among DNA methylation, histone modifications and microRNA dysregulation, in both understanding cancers and developing cancer therapies.

## 5. Epigenetic Effects Associated with Carcinogenic Chemicals from Gasoline

The major epigenetic modifications (e.g., DNA methylation, histone acetylation) are susceptible to change by environmental stimuli [99,104,227,228], likely bridging the gaps between human cells and their microenvironments. This offers possible explanations for how intercellular factors change intracellular epigenetic landscape, altering tumor behaviors and increasing cancer risk. Carcinogenesis is a stepwise process of accumulation of genetic and epigenetic abnormalities that lead to such as malignant transformation. Although some cancer initiation and progression may be attributed to identifiable mutations in critical genes [229], a wide range of changes take place through largely unknown transitions. Mounting evidence suggests that initiation of carcinogenesis and cancerous lesions have an epigenetic basis [230], and the epigenetic alterations are equally important as the genetic mutations in transforming normal cells to tumor cells [231,232]. The key roles that the altered DNA methylation play in carcinogenesis as nongenotoxic mechanisms have been the subject of previous reviews [231,232]. This also includes the suggestion from the U.S. EPA’s proposed Cancer Risk Assessment Guideline, which refers explicitly to a role of DNA methylation aberrations in carcinogenesis.

**Figure 2:**
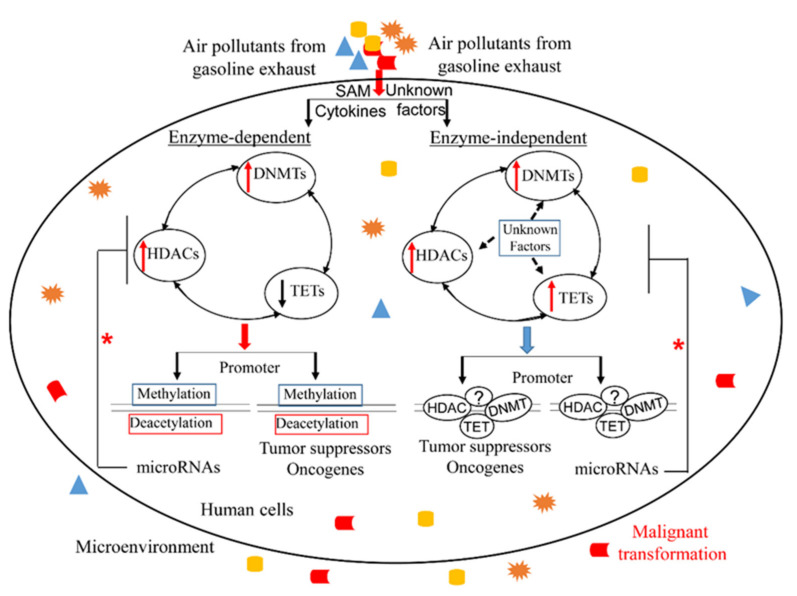
Air pollutants may induce malignant transformation via epigenetic aberrations. Chemicals from gasoline exhausts enter the human cells and modulate epigenetics through multiple mechanisms (e.g., abnormal levels of SAM and cytokine; unknown pathways), leading to malignant transformation. In human cells, left: HDACs, DNMTs and TETs cooperatively regulate DNA methylation and histone modification, thus changing target expression; right: HDACs, DNMTs, TETs and unknown factors (?) form complex binding target promoters, determining their levels; * microRNA deregulation feeds back to negatively modulate the expression of all epigenetic regulators.

Although many environmental factors, such as diet, lifestyles, therapeutic reagents, and chemicals, have been found to alter intracellular epigenetic signature, this review will focus on air pollutants emitting from gasoline combustion, including benzene, toluene, xylene, butadiene, 1,2,4–Trimethylbenzene, and 2–methylnaphthalene. In general, epidemiological and experimental studies support a carcinogenic potential of these chemicals, even at low doses [15,16,17,18,19,20,21,22]. Although genetic changes have been broadly studied in carcinogenic chemicals, as described in Figure 2, epigenetic alterations, due to their dynamic, rapid and reversible features, could be the first event followed by gene mutations leading to cancerous transformation. For instance, hypermethylation-silenced TSGs and hypomethylation-induced oncogene overexpression are plausible mechanisms that could underlie cancer initiation [233]. Mechanistically, promoters of DNA repair genes (e.g., MLH1, MGMT) can become methylated, which may lead to microsatellite instability and increased G-to-A transitions. Higher 5–mC content has higher potential to generate genetic mutations through the spontaneous deamination of 5–mC to thymine [234].

### 5.1. Benzene Induces Epigenetic Changes

Benzene is one major chemical from gasoline combustion and a demonstrated carcinogen. However, the mechanism underlying benzene-induced malignant transformation (e.g., hematotoxicity, lung cancer, breast cancer) has not been fully elucidated. Numerous in vitro and in vivo investigations revealed that benzene exposure modifies epigenetic marks, and most of these studies have centered on DNA methylation. Only a few studies have explored the contribution of environmental chemicals to changes in histone modifications and microRNA expression. This is supported by showing that alterations of DNA methylation patterns in normal and malignant cells mediate toxicity from benzene [24,36,235,236,237], including global DNA methylation as measured by DNA methylation changes in long interspersed nuclear element–1 (LINE–1) and AluI repetitive elements (a significant reduction in LINE–1 and AluI; loss of global DNA methylation) [36], as well as gene-specific/promoter DNA methylation [238] [MAGE–1, p15, p16, ERCC3; poly(ADP–ribose) polymerases–1 (PARP–1); Hypermethylation in p15 and p16; hypomethylation in MAGE–1] [29,36,239,240,241], which leads to downregulation of genes with promoter DNA hypermethylation. Secondary to studies in DNA methylation is the investigation of histones showing that benzene exposure alters the histone protein (H4, H3) modifications, such as a decrease of histone acetylation and increase of histone lysine methylation (H3K4me3), at a global level and in the gene promoters (topoisomerase IIα (Topo IIα)) [31,236,242,243]. Concomitantly, histone deacetylase (HDAC) inhibitors, for instance trichostatin A and MCP30, are able to relieve benzene-induced hematotoxicity [31]. Although not as frequent as changes in DNA methylation and histone acetylation, exposure to benzene also dysregulates microRNAs in vitro and in vivo, serving as a possible biomarker to manage benzene exposure [244,245,246,247].

There are several possible reasons why benzene exposure alters DNA methylation in human cells. First, benzene enhances nitric oxide production in the bone marrow, thus inducing a posttranscriptional increase in DNMT activities. Second, reactive oxygen species and oxidative DNA damage produced by benzene may reduce binding affinity of the methyl–CpG binding protein 2 (MBD2), thereby changing 5mC levels. Third, DNA strand breaks induced by benzene exposure may increase DNMT binding affinity at specific sites. Fourth, a significant decrease in mRNA levels of DNA methyltransferase (DNMT) 1, 3a, 3b and MBD2 was observed post exposure to benzene alone or BTX [19,248], particularly benzene-induced DNMT3b upregulation [32]. It is still largely unclear how benzene changes DNMT gene expression. Although Rothman et al. did not find changes in IL–6 levels in peripheral blood from workers exposed to benzene [249], Gillis et al. did show that benzene metabolites can stimulate the production of chemokines, the proinflammatory cytokines TNF–alpha and IL–6, and the Th2 cytokines IL–4 and IL–5 [250]. Given our studies showing that IL–6 is a key regulator in DNMT1 gene expression [97,109], benzene-altered DNMT expression may take place through abnormal IL–6 production, which warrants comprehensive studies. Few studies are found to investigate the impacts of benzene on the expression of HDACs and ten–eleven translocation methylcytosine dioxygenases as well as the physical and functional interactions among HDACs, DNMTs and ten–eleven translocation methylcytosine dioxygenases in cancerous transformation.

### 5.2. The Impacts of Toluene, Xylene, 1,3–Butadiene, 1,2,4–Trimethylbenzene and 2–Methylnaphthalene on Epigenetics in Cancers

Although toluene, xylene and butadiene are major chemicals emitting from gasoline combustion, compared with benzene, far fewer experiments have been conducted to examine their potential to be carcinogens and their regulatory roles in aberrant epigenetics. It has been shown that exposure to BTX, VOCs, BTEX or TEX containing toluene and xylene changes microRNA expression and DNA methylation patterning [19,251,252,253]. However, it is difficult to exclude the impacts from benzene. As a single agent, exposure to toluene, even at low levels, has been found to change DNA methylation levels [254,255,256]. Hong et al. showed that twenty-six genes are upregulated and hypomethylated, while 32 genes are downregulated and hypermethylated using in vivo samples [254]; changes in histone modifications (acetylation pattern of histones H3 and H4) [257,258] and microRNA expression are also observed [251]. For instance, Lim et al. found 54 differentially expressed microRNAs in HL–60 cells and exosomes upon toluene exposure [252]. Further, xylene exposure is reported to alter gene expression and DNA methylation [254,259,260]. In addition, 1,3–Butadiene (BD) is a common environmental pollutant that is classified as carcinogenic to humans. Studies also showed that exposure to BD changes DNA methylation and/or histone methylation in mouse models [15]. Notably, all these findings result from occupational exposure, but not mimicking studies from gasoline combustion exposure. Few studies have observed changes in microRNA levels and expression of DNA methyltransferase, histone deacetylase, or ten–eleven translocation methylcytosine dioxygenase genes post exposure to toluene, xylene, and 1,3–butadiene.

### 5.3. Polycyclic Aromatic Hydrocarbons (PAHs)

PAHs are common air pollutants resulting from incomplete combustion of organic materials (e.g., fossil fuels), which contain two or more fused benzene rings arranged in various configurations. They are well-documented genotoxicants and potential carcinogens. Increasing evidence suggests that prenatal exposure to PAHs reduces global genomic methylation [261,262,263,264]. This is further supported by showing that changes in gene-specific and global DNA methylation may be the causative mechanisms of PAH-related health effects [265,266,267]. The methylation of key genes related to breast cancer, like retinoic acid receptor beta (RARβ) and adenomatosis polyposis coli tumor suppressor (APC), has been associated with the presence of PAH adducts in breast tumor tissue and various sources of PAH exposure [268,269]. Additional genes that display altered DNA methylation due to PAH exposures play a role in insulin resistance [270] and cancer [267]. All the genes described above show PAHs-related DNA hypermethylation, suggesting a potential role for PAHs as an environmental factor that can silence gene expression via epigenetic mechanism at site-specific loci. Consistently, Yang et al. [267] reported that PAH exposure induces CpG site-specific hypermethylation of the p16(INK4α) gene. The degree of p16(INK4α) methylation is related to the levels of internal exposure. Thus, p16(INK4α) hypermethylation might serve as an important biomarker for PAHs exposure and for early cancer diagnosis. The studies of PAH-mediated DNA methylation highlight the impacts of hydrocarbon mixtures, supporting a role for the aberrant epigenome in PAH-associated carcinogenicity. In addition, Zhang et al. reported that H3K36me3 can be an indicator of PAH exposure [271], and microRNA expression is changed upon PAH exposure [272,273]. However, no studies have been found to address whether PAH exposure changes the expression of DNA methyltransferase, histone deacetylases and/or ten–eleven translocation methylcytosine dioxygenase genes, which warrant systematic investigations.

## 6. Conclusions and Outlook

The review aims to summarize the evidence for a contributory role of air pollutants emitting from gasoline combustion to cancer burden and the molecular mechanisms involved with a focus on aberrant epigenetics. Epidemiological and experimental data support that exposure to benzene, toluene, xylene, butadiene and/or PAHs may increase the risk of cancer development and promote cancer growth and metastasis. For benzene, the evidence could be classified as sufficient and consistent; for toluene, xylene, butadiene, or PAHs, further in vitro and in vivo studies are necessary to make an accurate conclusion. Increasing evidence supports that blending ethanol into gasoline (biofuels) reduces emissions of toxic chemicals including secondary aromatics. It can be concluded that ethanol blending in gasoline is beneficial to human health, given that toxic/carcinogenic chemicals are significantly reduced due to displacement by ethanol. Mechanistically, we conclude that benzene exposure alters global and gene-specific DNA methylation as well as the expression of DNA methyltransferases, histone deacetylases or microRNAs in human normal and cancer cells, with a suggestive conclusion that other chemicals in gasoline have similar impacts. Regarding the histone modifications, tentative conclusions could be made that exposure to benzene, toluene, xylene, butadiene and/or PAHs changes histone acetylation and lysine methylation. Given that blending ethanol into gasoline reduces chemicals that induce DNA methylation aberrations, biofuels could have positive effects on reducing cancer burden through restoring key antineoplastic features of aberrant epigenome in human cells.

Importantly, whether and how inhalation of ethanol vapor or exhaust products is harmful to human health remains largely elusive. Given the difficulty in mimicking ethanol inhalation from vapor or exhaust products, and because no reports are found to investigate the epigenetic effects of ethanol inhalation, we are unable to draw any definitive conclusions or make absolute comparisons between the epigenetic effects of the inhalation of ethanol exhaust emissions and gasoline exhaust emissions. There is a need for larger and longitudinal studies in vitro and in vivo, which mimic the real exposure to chemicals (particularly the concentrations/doses) from gasoline combustion, to demonstrate their biological and epigenetic potential. Further, because epigenetic controllers (DNA methyltransferases (DNMTs), histone deacetylases (HDACs), ten–eleven translocation methylcytosine dioxygenases (TETs), microRNAs) have functional and regulatory interplays, future studies need to put more efforts into focusing on the contributions of chemicals to the cooperative, but not individual, roles of these epigenetic controllers in cancer development. As gene dysregulation of epigenetic controllers occurs more frequently than mutations, more efforts are needed to address whether and how chemicals from gasoline combustion modulate the expression levels of DNMTs, HDACs and TETs. In addition to the classical epigenetic modifications (DNA methylation, histone acetylation), RNA/DNA N6–methyladenosine (m^6^A) represents a new epigenetic code, and critically regulates various biological processes [274,275,276,277], including cancers [278,279,280]. In vitro and in vivo studies are needed to investigate whether and how exposure to chemicals from engine exhaust regulates m^6^A levels and its modulators, which may change the rate of cancer initiation. Finally, given that epigenetic alterations are associated with viral infection, and because air pollutants from gasoline combustions induce epigenetic changes, studies focusing on the crosstalk among air pollutants, COVID-–19 and aberrant epigenetics should be initiated, which may find answers for why exposure to PM increases COVID-19 spread and transmission.

## Figures and Tables

**Table 1:** List of major compounds emitted from vehicle engine exhaust.

Acrolein	Inorganic Sulfates and Nitrates
Ammonia	Methane
Benzene	Methanol
1,3–Butadiene	Nitric acid
Carbon monoxide	Metals (e.g., lead and platinum)
Formaldehyde/Acetaldehyde	Nitrous acid
Formic acid	Nitrogen oxides
Heterocyclics and derivatives	Oxides of nitrogen
Hydrocarbons (C1–C18) and derivatives	Polycyclic aromatic hydrocarbons and Derivatives
Hydrocarbons (C14–C35) and derivatives	Sulfur oxides
Hydrogen cyanide	Toluene
Hydrogen sulfide	Nitrated hydrocarbons

## Abbreviations

BTX Butadiene, Toluene, Xylene; BTEX Butadiene, Toluene, Ethylbenzene, Xylene; DNMT1 DNA Methyltransferase 1; DNMT3A DNA Methyltransferase 3A; DNMT3B DNA Methyltransferase 3B; DNMT3L DNA methyltransferase 3L; EPA Environmental Protection Agency; FABP4 Fatty Acid-Binding Protein 4; HATs Histone Acetyltransferases; HDACs Histone Deacetylases; LINE–1 Long Interspersed Nuclear Element–1; miRs MicroRNA; 5mC 5–Methylcytosine; 5hmC 5–Hydroxy–Methylcytosine; IARC International Agency for Research on Cancer; PARP–1 Poly(ADP–ribose) Polymerases–1; PM Particulate Matter; PMI Particulate Matter Index; PAHs Polycyclic Aromatic Hydrocarbons; SAM S–Adenosyl–methionine; TET1 Ten–Eleven Translocation Methylcytosine Dioxygenase 1; TET2 Ten–Eleven Translocation Methylcytosine Dioxygenase 2; TET3 Ten–Eleven Translocation Methylcytosine Dioxygenase 3; THC Total Hydrocarbons; Topo IIα Topoisomerase IIα; TSGs Tumor Suppressor Genes; VOCs Volatile Organic Compounds
